# Radikale pelvine Tumorchirurgie bei Patienten mit einem lokal fortgeschrittenen, symptomatischen kastrationsresistenten Prostatakarzinom

**DOI:** 10.1007/s00120-021-01619-0

**Published:** 2021-08-23

**Authors:** Saskia Kanzelmeyer, Mark Bludau, David Johannes Karl Paul Pfister, Axel Heidenreich

**Affiliations:** 1 Klinik und Poliklinik für Urologie, Uro-Onkologie, spezielle urologische und roboter-assistierte Chirurgie, Universitätsklinikum Köln, Kerpener Str. 62, 50937 Köln, Deutschland; 2 Klinik für Urologie, Medizinische Universität Wien, Wien, Österreich; 3 Klinik und Poliklinik für Allgemein‑, Viszeral‑, Tumor- und Transplantationschirurgie, Universitätsklinikum Köln, Köln, Deutschland

**Keywords:** Radikale Prostatektomie, Radikale Zystoprostatektomie, Vordere Exenteration, Hintere Exenteration, Palliative Chirurgie, Radical prostatectomy, Radical cystoprostatectomy, Anterior pelvic exenteration, Posterior pelvic exenteration, Palliative care

## Abstract

**Ziel der Arbeit:**

Die retrospektive Evaluation der klinischen Ergebnisse nach palliativer pelviner Tumorchirurgie (ppTC) bei subvesikalen und supravesikalen Komplikationen eines lokal symptomatisch fortgeschrittenen kastrationsresistenten (CRPC) Prostatakarzinoms.

**Patienten und Methoden:**

Insgesamt 84 Patienten mit lokal fortgeschrittenem und symptomatischem CRPC erhielten eine radikale Zystoprostatektomie (*n* = 71, 83,3 %) oder anteriore und posteriore Exenteration (*n* = 13, 16,7 %). Das lokale Staging erfolgte mittels MRT des Beckens, Zystoskopie und Rektoskopie. Ein systemisches Staging erfolgte mittels Computertomographie von Thorax, Abdomen, Becken sowie Skelettszintigraphie. Die perioperativen Komplikationen wurden nach Clavien-Dindo-Klassifikation evaluiert. Das primäre Studienziel war das symptomfreie Überleben (sÜL, Fehlen von Symptomen am unteren oder oberen Harntrakt, fehlende endoluminale oder perkutane Intervention).

**Ergebnisse:**

Nach einem medianen Follow-up von 43,5 (3–139) Monaten betrug das sÜL nach einem und drei Jahren 95,2 % bzw. 86,7 %. Insgesamt 86,7 % der Patienten blieben für ihre gesamte verbleibende Lebensdauer bezüglich lokaler Symptome beschwerdefrei. Das OS nach einem und drei Jahren betrug 92,9 % bzw. 54,7 %. Clavien-Dindo-Grad-2-, -3- und -4-Komplikationen ergaben sich in 19 (22,6 %), 7 (8,3 %) bzw. 3 (3,6 %) Patienten.

**Schlussfolgerungen:**

Die ppTC ist mit einer geringen Komplikationsrate möglich und führt zu deutlicher Symptomlinderung bei ca. 90 % der Patienten, von denen > 80 % für die verbleibende Lebenszeit beschwerdefrei verbleiben. Voraussetzung sind die Patientenauswahl, ein interdisziplinäres Vorgehen und eine entsprechende chirurgische Expertise.

## Abstract (en)

**Goal:**

The retrospective evaluation of clinical outcomes after palliative pelvic exenteration (PPE) in patients with subvesical and supravesical complications due to symptomatic locally advanced castration-resistant prostate cancer (CRPC).

**Patients and methods:**

In all, 84 patients with locally advanced and symptomatic CRPC underwent radical cystoprostatectomy (*n* = 71, 83.3%) or anterior and posterior exenteration (*n* = 13, 16.7%). Local staging was done via pelvic MRI (magnetic resonance imaging), cystoscopy, and rectoscopy. Systemic staging was performed by computed tomography of the thorax, abdomen pelvis, and skeletal scintigraphy. Perioperative complications were evaluated using the Clavien–Dindo classification. The primary study objective was symptom-free survival defined as absence of lower or upper urinary tract symptoms and absence of endoluminal or percutaneous intervention.

**Results:**

After a median follow-up of 43.5 (3–139) months, symptom-free survival at 1 and 3 years was 95.2% and 86.7%, respectively. In all, 86.7% of patients remained symptom-free for their remaining lifetime with respect to local symptoms. Overall survival at 1 and 3 years was 92.9% and 54.7%, respectively. Clavien-Dindo grade 2, 3, and 4 complications occurred in 19 (22.6%), 7 (8.3%), and 3 (3.6%) patients, respectively.

**Conclusion:**

With adequate patient selection, PPE is possible with a low complication rate and results in significant symptom relief in the lower or upper genitourinary tract in about 90% of patients, of whom more than 80% remain symptom-free for the remainder of their lives. Prerequisites for favorable surgical outcomes are patient selection, an interdisciplinary approach, and appropriate surgical expertise.

## Einleitung

Das Prostatakarzinom (PCA) kann bei lokal fortgeschrittenem, aggressivem Tumorwachstum nach primärer Lokaltherapie trotz initiierter systemischer medikamentöser Tumortherapie konsekutiv die Harnblase und/oder das Rektum infiltrieren [1]. Eine Infiltration dieser Organe geht häufig mit schwerwiegenden Symptomen wie rektalen Blutungen, Makrohämaturie ± Blasentamponade, Harnverhalt, perinealen Schmerzen oder Hydronephrose einher.

Die überwiegende Mehrzahl dieser Patienten wird konservativ symptomatisch mittels palliativer TURP, transurethraler oder suprapubischer Katheteranlage bzw. endoluminaler Harnleiterschienung oder perkutaner Nephrostomie behandelt. Entsprechend aktueller retrospektiver Untersuchungen gehen alle diese Maßnahmen trotz vorübergehender Symptomenreduktion mit einer Einschränkung der Lebensqualität einher [2]. Eine effektive Kontrolle der lokalen Symptomatik wirkt sich zwar nicht auf das Gesamtüberleben aus, geht aber mit einer signifikanten Verbesserung der Lebensqualität einher und ist somit als Therapieziel definiert [3, 4].

Die palliative, radikale pelvine Tumorchirurgie (ppTC) stellt eine interdisziplinäre Behandlungsmöglichkeit dar, die bei adäquat ausgewählten Patienten mit lokal fortgeschrittenem und symptomatischem kastrationsresistenten Prostatakarzinom (CRPC) mit einer langfristigen Symptomkontrolle einhergehen kann.

Wir berichten über ein Kollektiv von 84 Patienten, bei denen eine ppTC unterschiedlicher Ausdehnung durchgeführt wurde und wir analysierten das symptomfreie Überleben, das Gesamtüberleben sowie die perioperative Komplikationsrate.

## Patienten und Methoden

Es erfolgte eine retrospektive Analyse von 84 Patienten, die zwischen 2008 und 2021 bei lokal fortgeschrittenem, symptomatischem CRPC mittels ppTC an den jeweiligen Institutionen der Operateure behandelt wurden.

Die Indikation zur ppTC wurde bei symptomatischer Infiltration von Blase und/oder Rektum unter laufender systemischer Therapie bei Vorliegen signifikanter Symptome wie rezidivierende Harnverhaltungen, Makrohämaturien mit/ohne Blasentamponade oder Bluttransfusionen, schmerzhafte Beckenbodeninfiltration, rektaler Obstruktion mit signifikanten Defäkationsproblemen oder obstruktivem Ileus gestellt.

Einschlusskriterien für den operativen Eingriff waren: ECOG-Performance-Status von 0–1, ASA-Status ≤ 2, Lebenserwartung > 1 Jahr und Symptome der lokalen Progression (rezidivierende Makrohämaturien mit oder ohne Bluttransfusion, resistente Beckenschmerzsymptomatik, obstruktiver Ileus, rezidivierende Harnverhaltung, signifikante Obstruktion des oberen oder unteren Harntraktes) trotz fortlaufender systemischer medikamentöser Tumortherapie ohne Nachweis einer systemischen Progression.

Präoperativ wurden die folgenden Laborparameter evaluiert: PSA, Albumin, alkalische Phosphatase, Leber- und Nierenfunktionsparameter, C‑reaktives Protein (CRP), Hämoglobin (Hb), Leukozyten, Thrombozyten und Laktatdehydrogenase (LDH). Der Testosteronwert musste im Kastrationsniveau < 50 ng/dl gelegen sein.

Die Patienten erhielten zur Beurteilung der lokalen und systemischen Ausdehnung des CRPC eine umfassende Bildgebung mit Computertomographie von Thorax, Abdomen und Becken, Kernspintomographie der Prostata und des Beckens sowie Skelettszintigraphie. Eine ^68^Ga-PSMA-PET/CT-Untersuchung wurde aufgrund der fehlenden therapeutischen Konsequenzen nur bei 17/84 Patienten durchgeführt.

Im Falle Defäkationsbeschwerden erhielten alle Patienten eine Rektoskopie zum Ausschluss einer rektalen Infiltration.

Die radikale Zystoprostatektomie mit inkontinenter Harnableitung stellte Therapie der Wahl bei Infiltration der Harnblase dar (Tab. 1). Im Falle einer rektalen Tumorinfiltration erfolgte eine anteriore und posteriore Exenteration als En-bloc-Resektion mit inkontinenter Harnableitung und einer endständigen Kolostomie.

**Table 1:** 

Parameter	Patientenkollektiv (*n* = 83)
Mittleres Alter (Jahre)	69,2 (56–82)
*Primärtherapie*	
Radikale Prostatektomie	8 (9,5 %)
Perkutane Radiotherapie (72–81 Gy)	46 (54,8 %)
Androgendeprivation	30 (35,7 %)
*Sekundärtherapie*	
Salvage RT + ADT + API	34 (40,5 %)
ADT + API + Docetaxel	30 (35,7 %)
ADT + API + Zweitlinie API	20 (23,8 %)
*ECOG-Performance-Status*	
0	66 (78,6 %)
1	18 (21,4 %)
*Metastasenlokalisation*^*a*^	
Lymphknotenmetastasen	26 (30,9 %)
Viszerale Metastase	7 (8,3 %)
Ossäre Metastasen	59 (70,2 %)
nmCRPC	12 (14,3 %)
*Laborbefunde*	
PSA (ng/ml)	31,4 (9,8–157,3)
Testosteron (ng/dl)	29,4 (17,1–41,4)
Hämoglobin (g/dl)	11,3 (8,8–12,3)
LDH (U/l)	182,3 (89,1–324,5)
CRP (mg/l)	6,3 (< 5,0–67,2)

*RT* Strahlentherapie, *ADT* Androgendeprivation, *ARI* Andrognezeptorinhinbitor, *nmCRPC* nichtmetastasiertes kastrationsresistentes Prostatakarzinom, *LDH* Lactdehydrogenase, *CRP* C-reakitves Protein

^a^Mehrfachlokalisationen sind möglich

Positive Schnittränder wurden durch den Nachweis von PCA-Zellen in der markierten Oberfläche von Prostata, Blase und/oder Rektum definiert.

Alle Patienten erhielten peri- und postoperativ eine Antithromboseprophylaxe (Enoxaparin 40 mg/Tag, 21 Tage) und Antibiose mit Metronidazol (400 mg 2‑mal täglich, 3 Tage) und Cefuroxim (500 mg 3‑mal täglich, 7 Tage).

Gemäß unseres klinikinternen Standards nach Anlage eines IC wurden die eingelegten Ureterkatheter für 7 Tage belassen und erst bei Nachweis einer patenten ureteroilealen Anastomose via retrograder Darstellung entfernt.

Die perioperativen Komplikationen wurden mittels Clavien-Dindo klassifiziert [5].

Bei allen Patienten wurde die systemische medikamentöse Tumortherapie kontinuierlich fortgesetzt. Die tumorspezifische Nachsorge der Patienten umfasste typischerweise in 3‑monatlichen Intervallen: PSA-Wertkontrolle, körperliche Untersuchung und Sonographie des oberen Harntrakts. Bei signifikanter PSA-Progression wurde zur Ausbreitungsdiagnostik eine Bildgebung mittels CT Thorax, Abdomen, Becken sowie einer Skelettszintigraphie sowie ab 2018 in Einzelfällen mittels ^68^Ga-PSMA-PET/CT veranlasst.

Das symptomfreie Überleben (sÜL) wurde definiert als ein Fehlen von Symptomen am oberen oder unteren Harntrakt bzw. das Fehlen von endoluminalen oder perkutanen Interventionen.

### Statistik

Die statistische Auswertung erfolgte STATA 16 (Stata Corp., College Station, TX, USA).

Die Ergebnisse wurden als signifikant gewertet, wenn sich im zweiseitigen Test ein *p*-Wert < 0,05 errechnete. Kaplan-Meier-Analysen wurden verwendet, um das symptomfreie Überleben und das Gesamtüberleben zu evaluieren. Univariate und multivariate Cox-Regressionsanalysen wurden durchgeführt, um eine Assoziation zwischen ECOG-Performance-Status sowie präoperativen Laborparametern mit den perioperativen Ergebnissen zu evaluieren.

## Ergebnisse

Die Patientencharakteristika sind in Tab. 1 dargestellt. 8 bzw. 46 Patienten waren einer radikalen Prostatektomie bzw. einer perkutanen Strahlentherapie als primärer lokaler Therapie zugeführt worden, während 30 Patienten eine primäre ADT ohne lokale Therapie erhielten.

Alle Patienten wiesen ein CRPC auf (Serumtestosteronkonzentration < 50 ng/dl, konsekutiv ansteigende PSA-Werte, Progression in der bildgebenden Diagnostik). Folgende therapierefraktäre Symptome führten zur ppTC: subvesikale Obstruktion in 69 (82,1 %), supravesikale Obstruktion in 46 (54,8 %) bzw. eine Kombination aus beidem, rezidivierende Makrohämaturien mit Blasentamponaden und/oder Transfusionspflichtigkeit in 29 (34,5 %) bzw. ein obstruktiver Subileus/Ileus in 8 (9,5 %).

**Figure 1:**
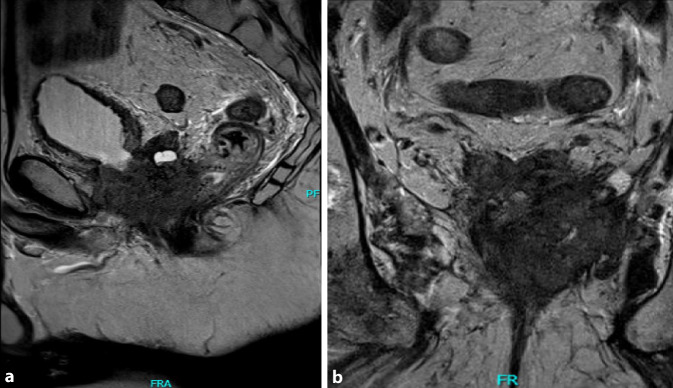

Das Ausmaß der lokalen Tumorausdehnung variierte zwischen einer Infiltration des Blasenhalses über die Infiltration der dorsalen Blasenwand bis hin zur Infiltration von Blase und Rektum (Abb. 1 und 2), sodass bei 71 bzw. 13 Patienten eine radikale Zystoprostatektomie mit Harnableitung bzw. eine totale Exenteration mit Harnableitung und Anlage eines Kolostomas erfolgte. Pathohistologisch zeigte sich bei allen Patienten ein Adenokarzinom der Prostata und in keinem Falle lag ein reines oder dominierendes neuroendokrines Prostatakarzinom vor.

**Figure 2:**
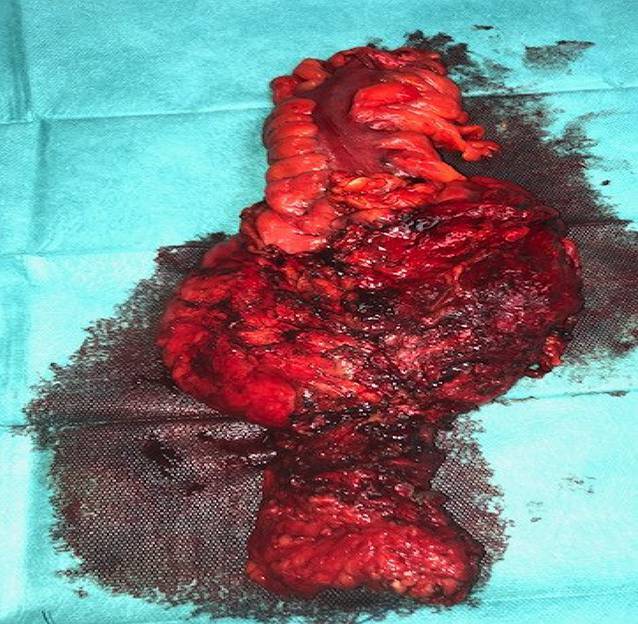

Zum Zeitpunkt der ppTC wiesen 7 (8,3 %) Patienten viszerale Metastasen auf, 65 (77,4 %) Patienten hatten retroperitoneale und/oder ossäre Metastasen und bei 12 (14,3 %) lag ein metastasenegatives, symptomatisches lokales Rezidiv mit einer PSA-Verdopplungszeit > 12 Monaten vor.

In Tab. 2 sind die perioperativen onkologischen und funktionellen Ergebnisse nach ppTC dargestellt. 83 % der Patienten wurden mittels radikaler Zystoprostatektomie und Anlage einer inkontinenten Harnableitung therapiert; bei 17 % der Patienten war eine anteriore und posteriore Exenteration erforderlich. Es traten keine Clavien-Dindo-Grad-5-Komplikationen auf. Clavien-Grad-2-, -3- und -4-Komplikationen entwickelten sich in 22,6 %, 8,3 % bzw. 3,6 % während der ersten 90 postoperativen Tage (Tab. 2). Der mittlere Hospitalisationsdauer betrug 20,7 (9–34) Tage. 6 Patienten (5,8 %) entwickelten perioperativ eine rapide Tumorprogression und verstarben innerhalb von 6 Monaten postoperativ. Alle diese Patienten hatten hohe CRP-Werte (Mittelwert 53,4 ± 12,3 mg/dl) und hohe PSA-Serumwerte (Mittelwert 95,6 ± 19,3 ng/ml).

**Table 2:** 

Variablen	*n* (%)
*Art der ppTC*	
Anteriore Exenteration	71 (83,3)
Totale Exenteration	13 (16,7)
*Art der Harnableitung*	
Ileumconduit	78 (92,8)
Ureterokutaneostomie	6 (7,2)
*Pathohistologisches Staging bei ppTC*	
pT3	10 (11,9)
pT4	74 (88,1)
pN0	2 (2,4)
pN+	21 (25)
pNx	61 (72,6)
R1	37 (44,1)
*Mittlere Operationszeit*	*271 (210–310) Minuten*
**Komplikationen**	
*Clavien-Grad 2*	*19 (22,6)*
*Clavien-Grad 3*	*7 (8,3)*
Wundrevision	3 (3,6)
Ureteroileale Anastomoseninsuffizienz, DJ-Stent	2 (2,4)
Fasziendehiszenz	2 (2,4)
*Clavien-Grad 4*	*3 (3,6)*
Lungenarterienembolie	1 (1,2)
Obstruktionsileus	1 (1,2)
Darmperforation	1 (1,2)

**Figure 3:**
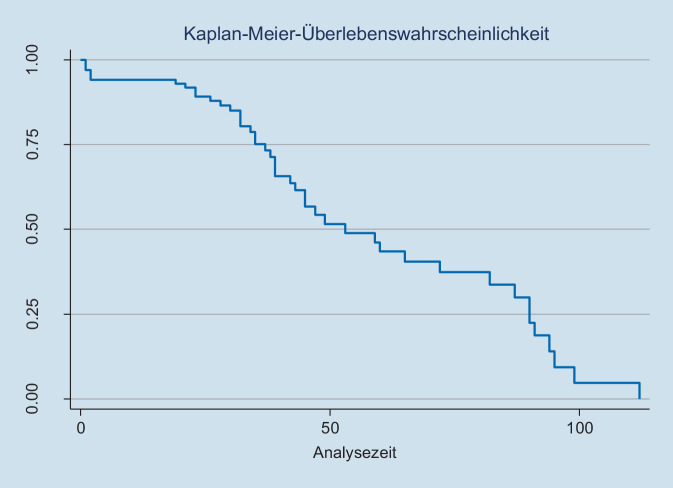

Bei einer medianen Nachbeobachtungszeit von 43,5 (3–139) Monaten verblieben 75 (89,2 %) Patienten symptomfrei. Das 1‑ und 3‑Jahres-SFS betrug 95,2 % bzw. 89,2 % (Abb. 3). 6 Patienten (5,8 %) benötigten aufgrund einer Obstruktion der oberen Harnwege durch progrediente retroperitoneale Lymphadenopathie (*n* = 4) oder Stenose der ureteroilealen Anastomose (*n* = 2) eine endoluminale Harnleiterschienung. Ein Patient entwickelte während der Nachsorge einen obstruktiven Ileus aufgrund einer Peritonealkarzinose und 2 weitere Patienten entwickelten einen obstruktiven Ileus aufgrund von Dünndarmadhäsionen. 38 (45,2 %) Patienten verstarben während der Nachbeobachtungsphase. Die Überlebensrate nach 1 Jahr sowie 3 Jahren betrug 92,9 % bzw. 54,7 %. Von den 46 Patienten waren nach 3 Jahren noch immer 42 (86,7 %) im Urogenitaltrakt symptomfrei.

## Diskussion

Trotz moderner lebensverlängernder und onkologisch effektiver Medikationen zur Behandlung des CRPC bleibt das lokal fortgeschrittene und symptomatische PCA eine therapeutische Herausforderung [6, 7]. Obwohl ADT mit oder ohne systemischer Kombinationstherapien die Progression von Systemmetastasen verlangsamen und kontrollieren können, sind ist eine intrapelvine Progression mit signifikanter Symptomatik nicht selten. Patrikidou et al. berichten, dass etwa zwei Drittel der Männer mit neu diagnostiziertem metastasiertem PCA unter lokoregionären Symptomen leiden, die in 60 % der Fälle chirurgische Interventionen wie die palliative TURP (36 %) oder endourologische Maßnahmen (25 %) erfordern [3]. Ähnliche Daten wurden von Won et al. angeführt [8], die eine Kohorte von 263 Patienten mit CRPC analysierten, von denen 45 % eine signifikante subvesikale Obstruktion oder Hydronephrose entwickelten, wenn der Primärtumor nicht operativ, sondern mit ADT oder ADT plus RT behandelt wurde. Diese Symptome haben zwar keinen Einfluss auf das Überleben, haben aber negative Auswirkungen auf die Lebensqualität. Aus et al. [9] zeigten, dass 61 % der CRPC-Patienten mindestens einmal in ihrer verbleibenden Lebenszeit palliativ operiert werden müssen, wenn der Primärtumor initial konservativ behandelt wurde. Die Patienten wurden in ihrem letzten Lebensjahr im Mittel 5 Wochen stationär zur Behandlung einer durch lokale Tumorprogression verursachten Symptomatik hospitalisiert. Auch in neueren prospektiven klinischen Studien wurde bei einem Drittel der Patienten ein symptomatischer Lokalprogress trotz Kombinationstherapie aus ADT, anderen lebensverlängernden Therapieoptionen und Radiatio der Prostata beobachtet [10]. Aufgrund der zunehmenden verbesserten medianen Überlebenszeiten von 4 bis 6 Jahren durch die modernen lebensverlängernden medikamentösen Therapiemaßnahmen ist zu erwarten, dass der Bedarf an chirurgischem Management von symptomatisch progredientem PCA zunehmen wird [11].

Die aktuelle Studie repräsentiert die größte Patientenkohorte, die sich einer ppTC bei symptomatischem, lokal progredientem CRPC unterzogen hat. Wir konnten zeigen, dass Patienten in gutem Allgemeinzustand (ECOG 0‑1, ASA 2), mit einer Lebenserwartung > 1 Jahr und ausschließlich lokalen Symptomen wenig signifikante operationsassoziierte Komplikationen erleiden und eine deutliche Symptomlinderung über mehr als 80 % der verbleibenden Lebenszeit erfahren. Jedoch verstarben 6 Patienten in den ersten 6 perioperativen Monaten aufgrund einer raschen systemischen Tumorprogression, die möglicherweise auf eine paraneoplastische inflamatorische Aktivierung des Immunsystems zurückzuführen sein könnte. Zwei retrospektive Studien wiesen in diesem Zusammenhang darauf hin, dass erhöhte CRP-Werte und erniedrigte Albuminserumkonzentrationen mit einem schlechteren progressionsfreien und Gesamtüberleben bei metastasiertem hormonsensitiven bzw. kastrationsresistenten PCA assoziiert sind [12–14]. Erhöhte CRP-Werte weisen auf eine Akute-Phase-Reaktion hin, die durch die Freisetzung von IL‑6 aus Prostatakrebszellen ausgelöst wird [13]. Auch wurde gezeigt, dass eine Erniedrigung des Albumins bzw. eine Erhöhung von CRP und IL‑6 mit dem erhöhten Risiko eines kurzfristigen Todes innerhalb der ersten 6 postoperativen Monate nach einer größeren onkologischen Operation verbunden sind [14]. Diese Patienten profitieren eher nicht von einer Operation, sondern benötigen eine sekundäre systemische Therapie inklusive einer optimalen palliativen und supportiven Behandlung.

Die Rate an Grad-4-Komplikationen nach ppTC lag bei 3,6 % und ist vergleichbar mit perioperativen Komplikationsraten anderer radikaler tumorchirurgischer Eingriffe im Becken, sodass die ppTC ebenfalls mit geringer Morbidität zu realisieren ist [15, 16]. Auch Grad-3-a–b-Komplikationen wurden nur in 8,3 % der Fälle beobachtet, was sowohl der strengen präoperativen Selektion der Patienten, als auch der chirurgischen Erfahrung geschuldet ist. Dennoch muss über eine Gesamtrate von 20 % signifikanter Komplikationen eine ausführliche präoperative Aufklärung erfolgen.

Bei der überwiegenden Mehrheit der Patienten konnte eine signifikante Reduktion der präoperativen Symptomatik für > 80 % der verbleibenden Lebenszeit erreicht werden. Die ppTC schaffte in dieser Patientenkohorte einen lang anhaltenden Nutzen durch effizientes Tumor-Debulking. Die Lebensqualität konnte durch das Fehlen von wiederholten Wechseln von Harnleiterschienen oder perkutanen Nephrostomien deutlich verbessert werden [17, 18].

Unsere Ergebnisse stehen im Einklang mit früheren Studien, die gezeigt haben, dass weder ADT noch Chemotherapie eine adäquate Symptomlinderung bei der Behandlung des lokal fortgeschrittenen und symptomatischen CRPC erreichen können [19–21]. Leibovici et al. demonstrierten bei 30 von 38 Patienten (88,2 %) mit lokal fortgeschrittener Tumorerkrankung eine signifikante und dauerhafte Reduktion der Symptome nach palliativer Zystoprostatektomie [19]. Kamat et al. berichteten über 14 Patienten, die aufgrund einer rektalen Invasion durch ein PCA unter Miktionsbeschwerden und pelviner Schmerzsymptomatik litten und nach Exenteration alle beschwerdegelindert waren [20]. Unsere Daten zeigten kürzlich bei einer Kohorte von 38 Patienten eine langfristige Symptomlinderung bei > 80 % der Patienten nach palliativer Zystektomie [21].

In allen Serien, einschließlich der unsrigen, variierten die Überlebensraten der Patienten nach ppTC zwischen unter 6 Monaten und mehreren Jahren. Dies verdeutlicht, dass die Patientenselektion von größter Bedeutung zu sein scheint, um Nutzen und Schaden der Operation abzuwägen und den palliativen Charakter der Maßnahme zu reflektieren.

Die palliative pelvine Strahlentherapie bei symptomatischem PCA kann als alternative Behandlungsoption zur ppTC bei Männern mit geringer lokoregionärer Tumorlast diskutiert werden [22, 23]. Allerdings wiesen alle von uns eingeschlossenen Patienten eine hohe Tumorlast auf, die durch eine RT nicht hätte kontrolliert werden können. Zwei Drittel der Patienten hatten sich bereits einer RT an der Prostata unterzogen.

Trotz der hohen Patientenzahlen ist unsere retrospektive Studie nicht frei von Limitationen. Die mittlere Nachbeobachtungszeit beträgt nur knapp 4 Jahre, wobei auch die begrenzte mittlere Gesamtüberlebenszeit bei Patienten mit CRPC berücksichtigt werden muss. Die positiven Ergebnisse könnten durch die oben beschriebene sorgfältige Patientenselektion verzerrt sein. Diese ist jedoch entscheidend, um signifikante behandlungsassoziierte Komplikationen, die die Lebensqualität beeinträchtigen oder sogar die Gesamtüberlebenszeit reduzieren könnten, zu vermeiden. Die fehlende konsequente Erfassung von Daten zur Lebensqualität mittels validierter Fragebögen stellt eine zusätzliche Limitation dar, da wir uns initial nur auf die Dokumentation der Symptomverbesserung fokussiert hatten.

## Fazit für die Praxis

- Unsere Studie repräsentiert die größte Kohorte von Patienten, bei denen eine palliative pelvine Exenteration bei lokal fortgeschrittenem, symptomatischem kastrationsresistentem Prostatakarzinom (CRPC) durchgeführt wurde. Sie ist daher von besonderer Bedeutung und liefert klinisch wichtige Informationen für das Management dieser Patienten.
- Die präoperative Patientenselektion ist entscheidend, nur Patienten in gutem Allgemeinzustand, mit Low-volume-Metastasierung und normalen Albumin- und CRP-Serumkonzentrationen sollten hinsichtlich dieser Art der Operation beraten werden. Diese genannten Parameter sind jedoch streng zu beachten, sodass nur ein sehr ausgewähltes Patientenkollektiv für die ausgedehnte Resektion in Frage kommt.
- Der Nutzen ist hoch, mit einem symptomfreien Überleben > 80 % der verbleibenden Lebenszeit. Unserer Erfahrung nach sollte die palliative pelvine Chirurgie mit radikaler Zystoprostatektomie und/oder posteriorer Exenteration als therapeutische Möglichkeit zur Symptomlinderung bei lokal fortgeschrittenem und symptomatischem CRPC standardisiert werden.
